# Urban Warming Drives Insect Pest Abundance on Street Trees

**DOI:** 10.1371/journal.pone.0059687

**Published:** 2013-03-27

**Authors:** Emily K. Meineke, Robert R. Dunn, Joseph O. Sexton, Steven D. Frank

**Affiliations:** 1 Department of Entomology, North Carolina State University, Raleigh, North Carolina, United States of America; 2 Department of Biology, North Carolina State University, Raleigh, North Carolina, United States of America; 3 Department of Geography, University of Maryland, College Park, Maryland, United States of America; DOE Pacific Northwest National Laboratory, United States of America

## Abstract

Cities profoundly alter biological communities, favoring some species over others, though the mechanisms that govern these changes are largely unknown. Herbivorous arthropod pests are often more abundant in urban than in rural areas, and urban outbreaks have been attributed to reduced control by predators and parasitoids and to increased susceptibility of stressed urban plants. These hypotheses, however, leave many outbreaks unexplained and fail to predict variation in pest abundance within cities. Here we show that the abundance of a common insect pest is positively related to temperature even when controlling for other habitat characteristics. The scale insect *Parthenolecanium quercifex* was 13 times more abundant on willow oak trees in the hottest parts of Raleigh, NC, in the southeastern United States, than in cooler areas, though parasitism rates were similar. We further separated the effects of heat from those of natural enemies and plant quality in a greenhouse reciprocal transplant experiment. *P. quercifex* collected from hot urban trees became more abundant in hot greenhouses than in cool greenhouses, whereas the abundance of *P. quercifex* collected from cooler urban trees remained low in hot and cool greenhouses. *Parthenolecanium quercifex* living in urban hot spots succeed with warming, and they do so because some demes have either acclimatized or adapted to high temperatures. Our results provide the first evidence that heat can be a key driver of insect pest outbreaks on urban trees. Since urban warming is similar in magnitude to global warming predicted in the next 50 years, pest abundance on city trees may foreshadow widespread outbreaks as natural forests also grow warmer.

## Introduction

Urban areas are generally hotter than surrounding rural areas [1]. This “urban heat island effect” results from the presence of less vegetation cover [2] and greater impervious surface cover [3] in cities compared to rural or natural areas [1]. Although urban warming was first noted in 1833 [4], the effects of heat on animal abundance and community characteristics in cities remain largely unknown. Instead, studies have emphasized the roles of habitat connectivity [5], [6] and resource availability [1], [7] in shaping urban animal communities. The effects of temperature deserve further attention because urban warming is becoming more extensive and more extreme as cities grow larger and is now coupled with global warming [8].

High urban temperatures should have the most pronounced effects on ectotherms, because thermal accumulation drives development in many ectothermic species [9]. Insects are of particular interest as the most diverse ectothermic taxon and because of their ecological and economic importance as pollinators [10], disease vectors [11], and plant pests [12]. Herbivorous insect pests are often more abundant in urban than in rural areas, though the proposed mechanisms for this pattern–changes in host plant quality [13], [14] and natural enemy efficacy [15]–do not consistently explain higher herbivorous insect pest abundance [16]. We hypothesize that the urban heat island effect is the most important driver of higher insect pest abundance in cities.

To test this hypothesis, we investigated the effects of urban warming on the biology of the soft scale insect *Parthenolecanium quercifex*. As a group, scale insects are among the most important pests of forest and landscape trees and are closely related to many other pests such as aphids and whiteflies. They are also sedentary and, thus, subject to the full effects of urban warming. We therefore selected *P. quercifex*, a common scale insect pest of oaks, as a study organism to test four specific hypotheses. First, we expected urban warming to increase *P. quercifex* abundance. Our approach to testing this hypothesis differs from that of other studies because we sampled scale insects on warm and cold trees within the city rather than comparing urban to surrounding rural areas [17], [7]. Second, we hypothesized that urban warming increases *P. quercifex* abundance by decreasing parasitism. To test this hypothesis, we measured percent parasitism [18] of *P. quercifex* in hot and cold sites. Third, we tested the hypothesis that urban warming increases *P. quercifex* abundance by increasing *P. quercifex* fecundity. This is a common physiological response to warming in ectotherms [19], [20], at least when warming pushes them toward their thermal optimum rather than beyond it [21]. Finally, we hypothesized that *P. quercifex* response to warming depends on thermal origin, such that *P. quercifex* from warmer areas have a physiological or adaptive advantage over individuals from cooler areas when placed in hot conditions. To test this hypothesis, we collected *P. quercifex* from warmer and cooler urban environments and placed them in warmer and cooler greenhouses. Because this common garden experiment provided trees with equal water and nutrients, we controlled for host plant quality, the other most common hypothesis for why herbivorous insect pests are more abundant in urban than in rural areas.

## Methods

### Study Organism

Soft scale insects (Hemiptera: Coccidae) are phloem-feeders on perennial plants [22]. They are commonly more abundant in cities than in rural areas [15], [16]. *Parthenolecanium quercifex* is an oak pest that has one generation per year and is native to North Carolina and much of North America [22]. Adults produce eggs in the late spring, usually in May [23]. Gravid females lay a dozen to several thousand eggs in an ovisac [22]. First instars migrate from ovisacs to leaves and feed on phloem throughout summer [22], [23]. In fall they molt and migrate back to tree stems [23]. Second instars overwinter and undergo development into adults in the early spring [23].

### Study Location

Raleigh has a humid subtropical climate, and the city center is located at 35.772096°N 78.638614°W. The average long-term winter temperature is 5.8°C. The average long-term summer temperature is 25.6°C. The average annual rainfall is 116.9 cm. Climate data were retrieved from the NOAA National Climatic Data Center (NCDC) (www.ncdc.noaa.gov) from the North Carolina State University weather station as 1981–2010 station normals.

### Hypothesis 1) Urban Warming Increases *P. quercifex* Abundance

We used thermal maps overlaid with maps of willow oak locations in ArcMap (ArcGIS Desktop 10, Redlands, CA) to locate study sites. To create thermal maps, winter and summer temperature measurements of the study area were extracted from the 120-m thermal band (Band 6) of Landsat-5 World Reference System 2 (WRS-2) path 16, row 35 images acquired on December 12, 2005 (winter) and August 18, 2007 (summer). The summer and winter multi-spectral images were geometrically rectified by polynomial transformation with nearest-neighbor resampling to 1-meter resolution, panchromatic digital orthorectified photographs acquired in March and April 1993, archived by the North Carolina Department of Transportation. The thermal-band images were then converted from 8-bit storage values to at-satellite brightness temperature (°C). Clouds and snow were identified visually using combinations of all seven spectral bands and removed manually.

**Figure 1:**
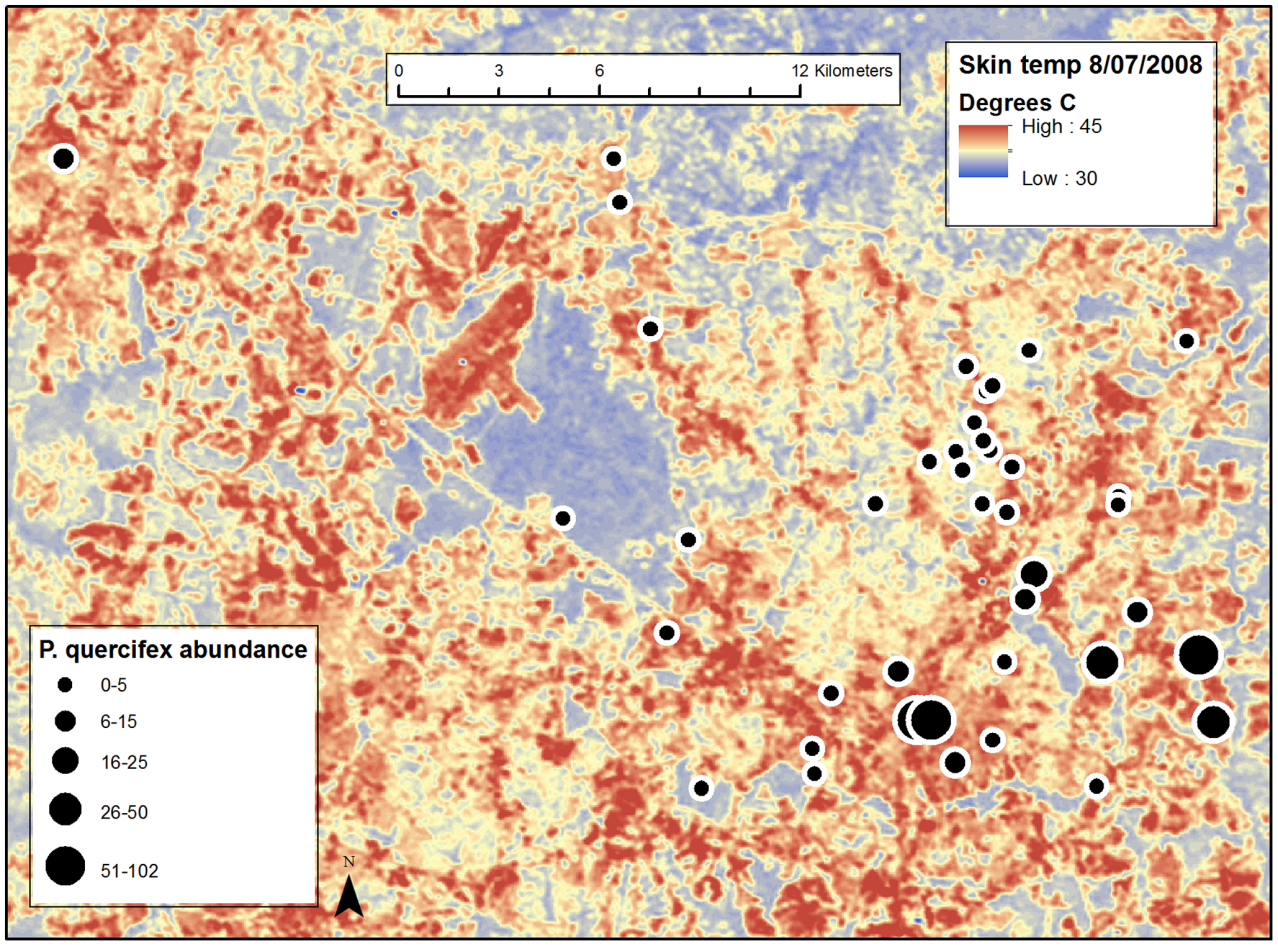
Thermal image overlaid with *Parthenolecanium quercifex* abundance across the Raleigh, NC urban heat island. 2^nd^ instar *P. quercifex* abundance across the Raleigh, NC urban heat island. Dots represent relative 2^nd^ instar *P. quercifex* abundance per 30.5 cm stem at each hot (red) and cold (blue) site (n = 40) in winter 2011. The image is a thermal map of the Raleigh, NC urban heat island created from a Landsat image acquired on August 18, 2007.

We identified 20 of the hottest (“hot”) and 20 of the coldest (“cold”) sites with at least two willow oak trees (Figure 1) in Durham, NC (1 site) and Raleigh, NC (39 sites). All sites were located in urbanized locations to minimize habitat related differences in natural enemy communities and host plant quality that might affect scale abundance. Each site was at least 200 meters away from any other site. This study was approved by the Raleigh Parks and Recreation Department, and all sites were located on public land except one site, which was located at a residence. Here, sampling was permitted by the homeowner. Sampling at all other sites was approved by the Raleigh Parks and Recreation Department.

We sampled 2^nd^ instar scale insects by collecting terminal 30.5 cm branches from each cardinal direction of study trees in January and February 2011 using a pole pruner. In the laboratory we counted 2^nd^ instar *P. quercifex* using a dissecting scope. We calculated mean scale insect abundance per branch on each tree. We then summed these values and divided them by the number of trees at each site (2) to generate a single insect-per-branch abundance value for each site. We compared mean scale abundance hot and cold sites with a t-test in SAS (SAS 9.1, Cary, NC).

Between April 20th and 29th, 2011, we sampled *P. quercifex* ovisacs by collecting the terminal 30.5 cm of one branch per tree at 6 hot sites and 5 cold sites (12 hot trees and 10 cold trees). To choose our study trees, we randomly selected individuals from the subset of trees occupied by 2^nd^ instar *P. quercifex* in our first sample. We selected trees occupied by *P. quercifex* to be sure higher abundance was due to differences in population growth rather than differences in colonization between hot and cold sites. Data did not meet ANOVA assumptions, even after log transformation with log(x+1), so we compared ovisac abundance per 30.5 cm between hot and cold sites with a Kruskal-Wallis Test in SAS (SAS 9.1, Cary, NC).

Between May 20th and 25th, 2011, we sampled 1^st^ instar scales on the same trees from which we sampled ovisacs by counting individuals on 10 leaves per study tree. We calculated mean abundance per 10 leaves on the two trees at each site. We compared log(x+1) transformed mean 1^st^ instar abundance on 10 leaves between hot and cold sites with a t-test in SAS (SAS 9.1, Cary, NC).

To measure temperature differences between hot and cold sites, we placed ibutton thermachrons (Dallas Semiconductor of Dallas, TX) that recorded temperature 6 times per day at a subset of sites (5 hot, 6 cold). We placed thermachrons in ibutton wall mounts (Dallas Semiconductor of Dallas, TX) inside a 2.54-cm deep plastic cup to protect them from precipitation and direct sun. Thermachrons were in place from May until August 2011. We calculated daily mean and maximum temperatures in each treatment. We then compared average mean and average maximum daily temperatures at hot and cold sites using a repeated measures ANOVA in SAS (SAS 9.1, Cary, NC).

### Hypothesis 2) Urban Warming Increases *P. quercifex* Abundance by Decreasing Parasitism

To test for the influence of warming on parasitoids and subsequent effects of parasitism on *P. quercifex* abundance, we collected one branch with 20 or more *P. quercifex* individuals from the same trees from which we sampled 1^st^ instars and ovisacs on five sampling dates while the scale were developing and laying eggs (March 7, April 22, April 29, May 20, and May 27, 2011). We dissected 20 individuals per branch for parasitoid larvae and marked each individual as parasitized or not parasitized. We calculated mean percent parasitism at each site on each date. We compared mean percent parasitism between hot and cold sites using a repeated measures ANOVA in SAS (SAS 9.1, Cary, NC). To identify parasitoids that attack *P. quercifex* in Raleigh, we clipped *P. quercifex* infested branches, removed all other arthropods, and placed them in cotton-plugged vials on each date. We reared out parasitoids from March to August 2012 in an incubator at 23°C, 50% humidity, and a 12 hr/12 hr light-dark cycle. We point-mounted each parasitoid that emerged and identified it to genus or species.

### Hypothesis 3) Urban Warming Increases *P. quercifex* Abundance by Increasing *P. quercifex* Fecundity

To determine whether *P. quercifex* fecundity differed between hot and cold sites, we collected 2 ovisacs from the same trees used to assess ovisac and 1^st^ instar abundance on April 29^th^, 2011. Ovisacs were returned to the laboratory in a cooler within 2 hours of collection. We emptied the eggs from each ovisac into a separate petri dish filled with 10 ml of 80% ethanol. We took a picture of each petri dish containing eggs using a Canon EOS DS126071 Rebel XT camera with a Canon EF-S 60-mm Macro lens. We used ImageJ (ImageJ 1.45 m, Bethesda, MD) to count the particles (eggs) in each image and the total area of those particles. To avoid counting multiple eggs as one, we used Image J to calculate the areas of ten eggs, found the mean of those areas, and divided the total egg area in each petri dish by the mean area of a single egg to get an egg count for each ovisac. We calculated mean egg counts for each ovisac at each site. Then we calculated mean egg count per tree and mean egg count per site. We compared mean egg count per ovisac between hot and cold sites with a t-test in SAS (SAS 9.1, Cary, NC).

### Hypothesis 4) *Parthenolecanium quercifex* Response to Warming Depends on Thermal Origin

To further isolate the effects of temperature from other biotic and abiotic effects on *P. quercifex* abundance and to test how *P. quercifex* origin affects response to temperature, we conducted a common garden experiment with a 2 by 2 factorial design, wherein we reared scales originating from hot and cold sites in hot (36°C day–18∶00–6∶00/32°C night–6∶00–18∶00) and cold (32°C day–18∶00–6∶00/28°C night–6∶00–18∶00) greenhouses. When scale matured in April 2011, we collected 4 ovisacs from a subset of our study trees (10 hot and 10 cold). We attached two ovisacs to each of 40 willow oak saplings in greenhouses at the NCSU phytotron facility in the two temperature treatments. Bare root willow oak saplings (1.04±0.02 m) were purchased from Rennerwood, Inc (Tennessee Colony, TX) and grown in 20.3 cm pots in Fafard 2P potting mix (Agawam, MA). They were fertilized 3 times per week with nutrient solution (N-P-K 10.2-1-10.7) mixed in the NCSU phytotron (http://www.ncsu.edu/phytotron/manual.pdf, pp. 15–16) and watered once per day. The potting media in both treatments was kept moist to ensure that high temperature did not result in water deficiency. Two weeks before infestation, saplings were treated with Tau Fluvalinate (Mavrik, Aquaflow) 1 mL/L H_2_0 to ensure no other insects were being transported into the greenhouses.

After egg hatch in April 2011, we counted settled first instar nymphs on 10 leaves per tree on May 10, 17, 26, and July 15, 2011. We used repeated measures ANOVA in SAS to compare 1^st^ instar abundance per 10 leaves among treatments.

## Results

### Hypothesis 1) Urban Warming Increases *P. quercifex* Abundance

**Figure 2:**
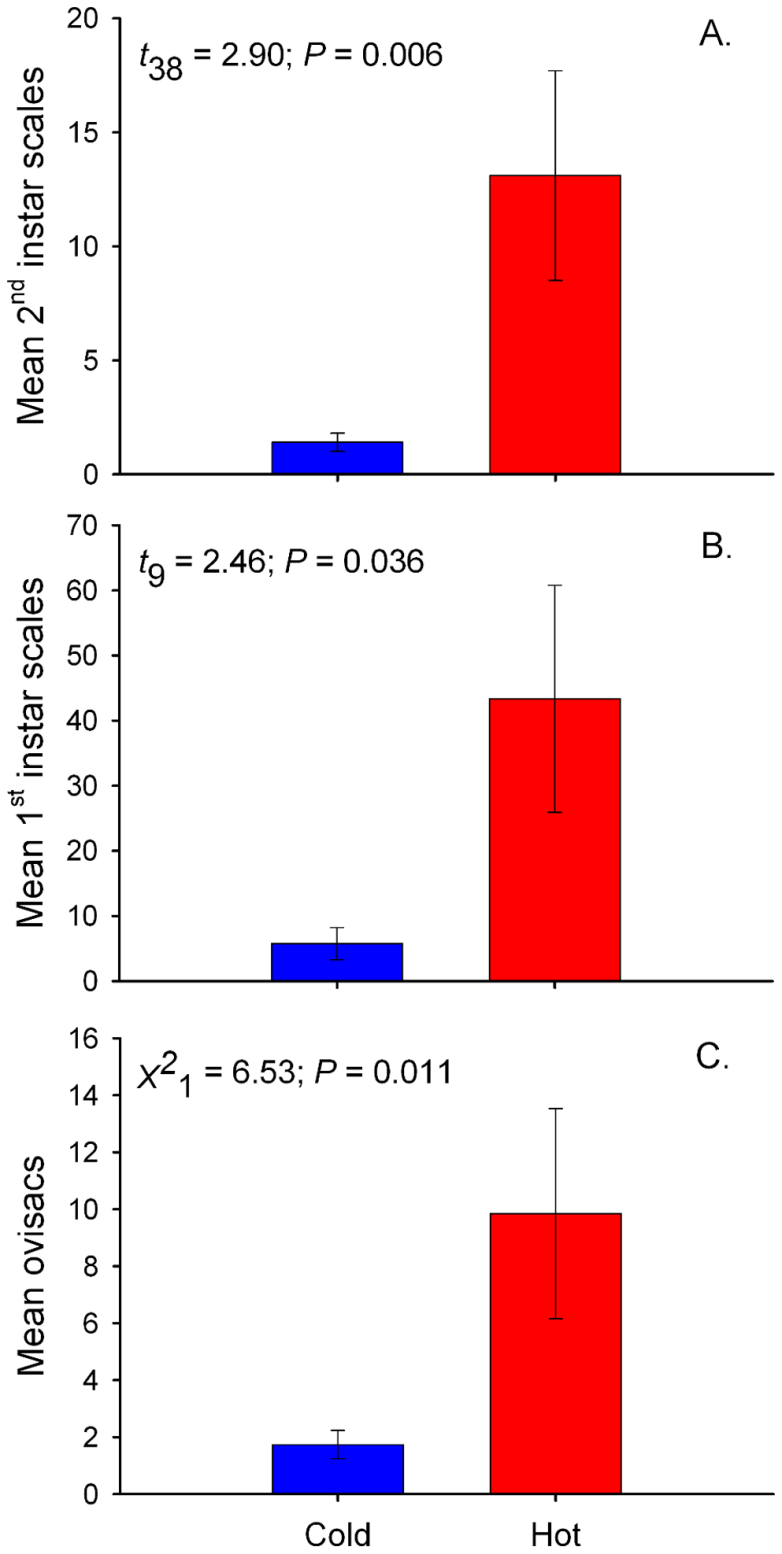
*Parthenolecanium quercifex* abundance across the Raleigh, NC urban heat island. Abundance of *P. quercifex* on hot and cold urban trees. Bars represent the mean (±SEM) abundance of (A) 2^nd^ instars in winter 2011 (n = 40); (B) 1^st^ instars in June 2011 (n = 11); and (C) ovisacs in spring 2011 (n = 11) on 30.5-cm terminal branches of hot (red) and cold (blue) urban trees in Raleigh, NC.

We found that overwintering second instars were 13 times more abundant on hot than on cold trees (*t_38_* = 2.90, *P* = 0.006; Figures 1 and 2A). In April 2011, ovisacs deposited by the same generation were 5.5 times more abundant on hot trees (*X^2^_1_* = 6.53, *P* = 0.011; Figure 2C). In June 2011, the next generation of 1^st^ instars was over 7 times more abundant on hot than cold trees (*t_9_* = 2.46, *P = *0.043; Figure 2B).

**Figure 3:**
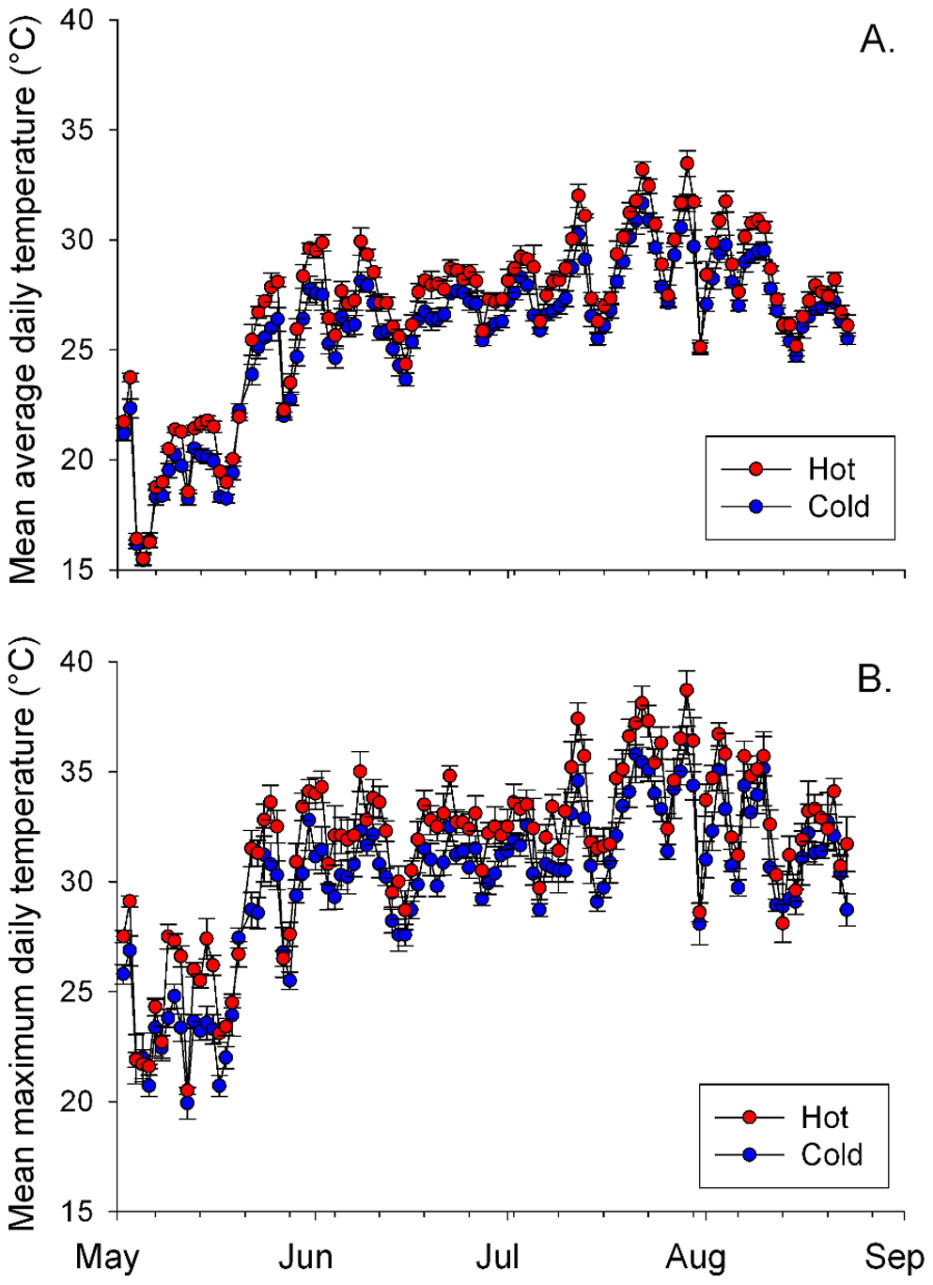
Average and maximum temperature differences between hot and cold sites. Temperatures recorded on ibuttons at ‘hot’ and ‘cold’ sites in Raleigh, NC May 2, 2011- August 23, 2011. Dots represent mean (±SEM) a) average daily temperature (°C) and b) mean maximum daily temperature at hot and cold sites. Average daily mean temperatures were significantly higher at hot sites (*F*_1, 10_ = 7.90, *P* = 0.0185), as were mean daily maximum temperatures (*F*_1, 10_ = 6.42, *P* = 0.0297). The extent of the difference between average (*F*_112, 1120_ = 1.96, *P*<0.0001) and maximum daily temperatures (*F*_112, 1120_ = 1.23, *P* = 0.0583) depended on time of year.

There was a significant interaction between site temperature and time, wherein the extent of the differences in mean average temperatures (*F*_112, 1120_ = 1.96, *P*<0.0001) between hot and cold sites depended on time of year. Similarly, the interaction between site temperature and time was marginally significant for mean maximum temperatures (*F*_112, 1120_ = 1.23, *P* = 0.0583). Mean average hot site temperatures were between 0–2.4°C higher than mean average temperature at cold site temperatures (*F*_1, 10_ = 7.90, *P* = 0.0185; Figure 3A), and mean maximum daily temperatures at hot sites were between 0–3.8°C warmer than mean maximum daily temperatures at cold sites (*F*_1, 10_ = 6.42, *P* = 0.0297; Figure 3B).

### Hypothesis 2) Urban Warming Increases *P. quercifex* Abundance by Decreasing Parasitism

**Figure 4:**
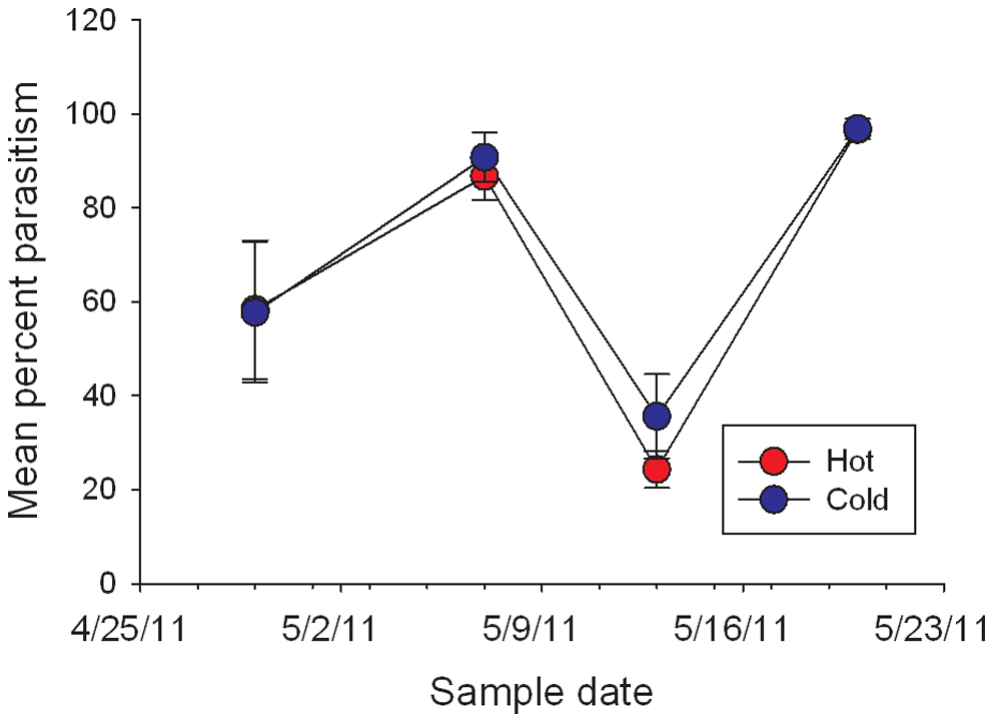
Percent parasitism of *P. quercifex* on hot and cold urban trees. Bars represent the mean (±SEM) percent of dissected 2^nd^ instars, adults, and ovisacs that had been parasitized on hot (red) and cold (blue) urban trees in Raleigh, NC on four dates in 2011. Temperature treatment had no significant effect on percent parasitism (*F*_1, 6.45_ = 0.21, *P* = 0.6631, n = 11).

We reared six parasitoid species from *P. quercifex*: *Coccophagus lycimnia* Walker (Hymenoptera: Aphelinidae), *Pachyneuron altiscutum* Howard (Hymenoptera: Pteromalidae), *Eunotus lividus* Ashmead (Hymenoptera: Pteromalidae), *Encyrtus fuscus* Howard (Hymenoptera: Encyrtidae), *Blastothrix* sp. Mayr (Hymenoptera: Encyrtidae), and *Metaphycus* sp. Mercet (Hymenoptera: Encyrtidae). Percent parasitism did not differ between *P. quercifex* from hot and cold sites (*F*_1, 6.45_ = 0.21, *P* = 0.6631; Figure 4).

### Hypothesis 3) Urban Warming Increases *P. quercifex* Abundance by Increasing *P. quercifex* Fecundity

The number of eggs in ovisacs from hot and cold sites did not differ (*t*_9_ = 1.87, *P* = 0.094).

### *Hypothesis 4) P. quercifex* Response to Warming Depends on Thermal Origin

**Figure 5:**
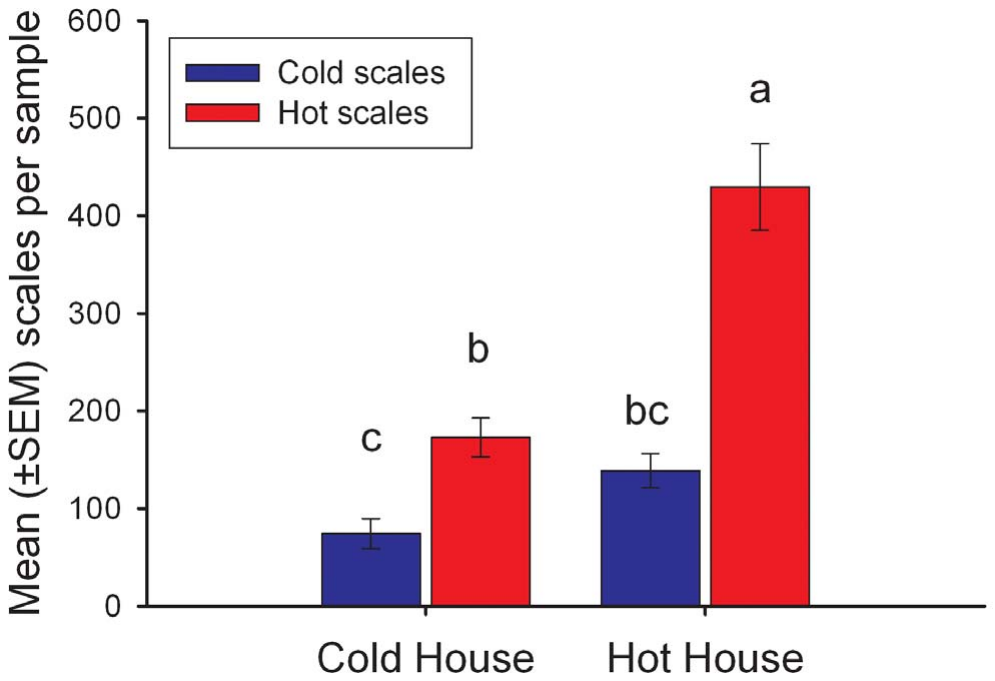
Abundance of *P. quercifex* in a common garden experiment. Bars represent the mean (±SEM) *P. quercifex* 1^st^ instars per 10 leaves. We calculated mean abundance across 4 sample dates (May 10, 17, 26, and July 15, 2011) since Repeated Measures ANOVA indicated there was no interaction of time with ovisac source or temperature treatment. 1^st^ instars hatched from ovisacs collected on hot (red) and cold (blue) urban trees, then reared on ‘hot’ and ‘cold’ greenhouse saplings (*x axis*). There was a significant interaction between *P. quercifex* ovisac source temperature and greenhouse temperature (*F*_1, 134_ = 11.57, *P* = 0.0009, n = 40). Detailed statistics are available in Table 1.

The effect of greenhouse temperature on scale abundance depended on scale origin, such that *P. quercifex* collected from hot trees reared in hot greenhouses were over twice as abundant as *P. quercifex* in any other treatment (*F*_1, 134_ = 11.57, *p-*value = 0.0009; Table 1, Figure 5). *P. quercifex* from cold trees did not become more abundant when reared in hot greenhouses. In the cold greenhouse, *P. quercifex* from hot trees were significantly more abundant than *P. quercifex* from cold trees; still, they were less than half as abundant as in hot greenhouses.

**Table 1:** Statistics for repeated measures ANOVA of *P. quercifex* abundance in common garden experiment. (An * denotes an interaction.).

Effect	Ndf, Ddf	*F*	*P*
Date	3, 134	0.35	0.7867
Source temp.	1, 134	46.57	<0.0001
Date* Source temp.	3, 134	0.04	0.9891
Greenhouse	1, 134	31.65	<0.0001
Date* Greenhouse	3, 134	0.67	0.5698
Source temp.* Greenhouse	1, 134	11.57	0.0009
Date* Source temp. * Greenhouse	3, 134	0.01	0.9987

## Discussion

We found urban warming directly leads to higher *P. quercifex* abundance. While the two most common hypotheses for elevated pest abundance in cities are changes in host plant quality and natural enemy efficacy [16], we found no evidence that either of these factors contribute to *P. quercifex* abundance patterns across the Raleigh, NC urban heat island. We also found no evidence that urban warming directly affects *P. quercifex* fecundity. Instead, we found evidence that *P. quercifex* populations may be locally adapted, or individuals acclimatized, to the temperature of the urban habitat patches in which they reside.

Urban trees are frequently stressed due to lack of water and nutrients [24], [25]. In some cases, stress can reduce tree defenses, leading to higher herbivore abundance [26]. Because our study sites were all in urban habitats, we have no reason to believe that nutrient levels available to trees covaried with temperature. It is conceivable that warm trees are more water stressed, and such a possibility deserves study. However, water stress tends to lead to decreases in the abundance of piercing-sucking herbivores [27], [28], which suggests that water stress should lead to lower *P. quercifex* abundance in hot urban areas. We observe the opposite pattern. Additionally, in our common garden experiment, we watered trees daily and provided equal nutrients to all trees to minimize any effects of water or nutrient stress. It is unlikely that differences in tree stress or quality account for the difference in scale abundance between hot and cold sites.

Natural enemies are often less abundant and diverse in urban than rural habitats. This difference has been cited to explain higher pest abundance in cities [16], [15]. All our study sites were within urban habitats, so–given that natural enemies tend to be relatively good dispersers [29], [30] –natural enemy communities should be similar among trees. As such, it is not surprising that we did not find a difference in percent parasitism between *P. quercifex* from hot and cold sites. Differences in parasitoid efficacy do not account for greater *P. quercifex* abundance on hot trees, as percent parasitism of *P. quercifex* on hot trees was equal to that of cold trees. Additionally, *P. quercifex* was more abundant in hot chambers in our greenhouse experiment, which excluded natural enemies. Thus, reduction of biological control by parasitoids does not explain high scale abundance at hot sites.

Our common garden experiment shows that *P. quercifex* is locally acclimated or adapted to urban thermal conditions and that this directly leads to higher abundance. *P. quercifex* from hot urban areas became almost 4 times more abundant than those from cold urban areas when placed in hot greenhouses. This effect is likely due to differences in survival, because we found no differences in fecundity between *P. quercifex* from hot and cold sites. We suggest that *P. quercifex* may locally adapt in response to urban warming, as other studies provide evidence for local adaptation in scale insects [31], [32]. The scale insect life cycle, which is often parthenogenetic and highly localized, inhibits gene flow [33], and evidence suggests this could lead to differentiation at small spatial scales [34]. Further, at least one other scale insects species has been shown to adapt to thermal conditions within its introduced range [35]. However, we cannot eliminate the possibility that observed abundance patterns resulted from maternal effects [36] or phenotypic plasticity of offspring leading to acclimation [37], rather than from genetic differences between *P. quercifex* from hotter and colder areas [38]. While the specific mechanism by which warming increases *P. quercifex* abundance warrants further investigation, our findings show that *P. quercifex* are primed to survive better in response to warming, be it urban or global.

For more than a century, scientists have documented that arthropod pests, including scale insects [39], are more abundant on urban trees than rural trees [16]. We provide evidence that urban heat may explain this effect, and we show that small temperature differences predict changes of an order of magnitude in pest abundance. We observed this effect over a temperature gradient common in many urban heat islands [1], indicating that urban warming poses a broad and immediate threat to urban trees and the services they provide, including cooling and carbon sequestration [2]. The adaptation or acclimation of herbivorous pests to warm environments may represent an ecological tipping point after which arthropod pests can overwhelm plant defenses and escape natural enemy control. Furthermore, temperature increases of similar magnitude are predicted under global climate change [40]. If rising global temperatures trigger an herbivore response similar to the one we observed in the city, then both urban and rural trees may be threatened by greatly increased herbivory in the future.
